# Ambulatory physiological measures obtained under naturalistic urban mobility conditions have acceptable reliability

**DOI:** 10.1038/s41598-025-13216-8

**Published:** 2025-08-07

**Authors:** Dilber Korkmaz, Kilian Knauth, Angela Brands, Marie Schmeck, Pia Büning, Jan Peters

**Affiliations:** Department of Psychology, Biological Psychology, University of Cologne, Cologne, Germany

**Keywords:** Ambulatory assessment, Reliability, Psychophysiological monitoring, Naturalistic environment, Urban real-world, Human behaviour, Neurophysiology, Health care

## Abstract

Ambulatory assessment methods in psychology and clinical neuroscience are powerful research tools for collecting data outside of the laboratory. These methods encompass physiological, behavioral, and self-report measures obtained while individuals navigate in real-world environments, thereby increasing the ecological validity of experimental approaches. Despite the recent increase in applications of ambulatory physiology, data on the reliability of these measures is still limited. To address this issue, twenty-six healthy participants (*N* = 15 female, 18–34 years) completed an urban walking route (distance *M* = 2.2 km, ±SD = 0.11; duration *M* = 30.8 min, ±SD = 1.34; temperature *M* = 18.34° degree Celsius, ±SD = 1.19, *Range* = 16°-21° degrees Celsius) on two separate testing days, while assessing the effect of metabolic state (sated vs. fasted). GPS-location and ambulatory physiological measures (cardiovascular and electrodermal activity) were continuously recorded. The results showed no significant differences in single physiological measures between fasted and sated states. Bootstrapped test-retest reliabilities of single measures and aggregate scores derived via principal component analysis (PCA) were computed. The first principal component (PC#1) accounted for 39–45% of variance across measures. PC#1 scores demonstrated an acceptable test-retest reliability (*r* = .60) across testing days, exceeding the reliabilities of most individual measures (heart rate: *r* = .53, heart rate variability: *r* = .50, skin conductance level: *r* = .53, no. of skin conductance responses: *r* = .28, skin conductance response amplitude: *r* = .60). Results confirm that ambulatory physiological measures recorded during naturalistic navigation in urban environments exhibit acceptable test-retest reliability, in particular when compound scores across physiological measures are analyzed, a prerequisite for applications in (clinical) psychology and digital health.

## Introduction

Ambulatory assessment utilizes diverse data sources to gain a deeper understanding of people’s thoughts, emotions, and actions in their natural environment^1^. The simultaneous acquisition of physiological, behavioral, and self-report data allows the study of individual behavior under natural conditions^2,3^. In contrast to traditional and highly controlled laboratory-centric approaches, ambulatory assessment methods can be systematically integrated into real-life environments through which individuals naturally navigate. This involves the use of portable devices and wearable sensors to capture continuous data streams in real-time^1^. Such approaches attempt to enhance ecological validity by allowing for the automatic assessment of environmental variables (e.g., temperatures, geolocations, time of the day^4^), while also capturing the temporal dynamics and contextual nuances of psychological and subjective experiences, as well as human behaviors. This minimizes potential biases associated with retrospective self-reports^5,6^. Furthermore, the constraint to laboratory settings poses challenges in transferring findings to real-world scenarios, potentially limiting the generalizability of observed psychophysiological responses^7^. By incorporating multimodal psychophysiological measures, these approaches can improve our understanding of the reciprocal interactions between psychological processes and physiological responses as they occur under natural conditions^8^.

Activity of the autonomic nervous system (ANS) as a proxy of the internal arousal state, plays a pivotal role in numerous behaviors and emotions^9,10^, operating independently of conscious effort and beyond voluntary control^11^. The ANS comprises two distinct components, the sympathetic nervous system (SNS) and the parasympathetic nervous system (PNS), which function as physiological antagonists, synergistically, or independently^12^. Commonly used physiological indices include cardiovascular activity (e.g. heart rate, heart rate variability), respiratory, and electrodermal activity (EDA). EDA is a direct measure of the current conductive properties of the skin^13^ and is closely linked to specifically SNS activity^13,14^. Heart rate variability, which measures variability in the time interval between successive heart beats, is closely related to PNS activity or vagal tone, although the precise association depends on the specific metric used for analysis^15,16^. For instance, Root Mean Square of Successive Differences (RMSSD) is more directly linked with vagal tone, while others, such as the Standard Deviation of NN intervals (SDNN), are thought to reflect both SNS and PNS activity^17^. Heart rate and skin conductance level tend to increase with increasing arousal. Concurrently, heart rate variability is being increasingly recognized as an indicator of autonomic flexibility and the ability to regulate emotions^15^. A combination of these at least partly complementary measures appears promising when aiming to comprehensively characterize the internal physiological response associated with emotional processing^18,19^, or self-regulatory mechanisms linked to cognitive, affective, social, and health phenomena^20,21^.

Various laboratory studies found that measures of ANS signaling convey objective information related to stimulus processing and decision-making^22–24^, that is not captured by subjective ratings^25,26^. Physiological measures can therefore offer valuable insights to (clinical) psychology^27–29^, and into how individuals respond to different contextual factors^26,30,31^. Exposure to natural environments, which influences emotional well-being and reduces stress^32,33^, is often evaluated in clinical studies using psychophysiological measures^34,35^. Evidence suggests that walking in urban green spaces has positive effects on psychosocial well-being^36,37^, mental restoration, cognitive function^38^, heart rate variability^39,40^, and brain activity^41^. Ambulatory psychophysiological assessment approaches also have potential utility for real-time monitoring and intervention in substance use disorders^35^.

Wearable sensor technologies are increasingly used to monitor a variety of physiological metrics in real-world settings, providing valuable insights into health and well-being. Some mobile sensors exhibit good reliability in laboratory conditions, supporting their application for continuous and unobtrusive physiological monitoring under real-life conditions^42^. It is important to note that while numerous studies have validated these devices under controlled laboratory and real-world settings, there can be discrepancies when they are used in everyday environments. Uncontrolled conditions of the real world often introduce additional variables that can affect sensor performance, leading to potential data loss and measurement inaccuracies (e.g. related to movement and environmental factors)^42^. The present research examines the reliability of physiological responses over time to determine whether the responses remain consistent in naturalistic settings, despite the known differences between laboratory and real-world environments.

Reliability assessments of psychophysiological metrics have so far largely focused on data obtained under controlled laboratory conditions. Here, heart rate and heart rate variability exhibit moderate to excellent reliabilities^23,43–45^. Likewise, lab-based EDA measures show moderate reliability^13,46–48^. In contrast, there is a dearth of studies investigating the reliability of ambulatory measures in naturalistic settings, particularly with regard to different physiological metrics. Although studies specifically focusing on the reliability of various physiological metrics in urban naturalistic contexts are lacking, some studies have reported the reliability estimates of physiological parameters during exposure to outdoor environments (e.g. nature viewing, outdoor walks, and outdoor exercise)^49^. Cardiovascular indices like heart rate and blood pressure have shown acceptable reliability during outdoor mobility^50–52^. However, there is a scarcity of evidence regarding the reliability of EDA measures in naturalistic conditions, as (to our best knowledge) no studies reporting reliability outcomes in outdoor environments have been found in the literature.

Bridging the gap between laboratory reliability and real-world settings, understanding how these metrics perform in naturalistic environments is critical to ensure the robustness of ambulatory psychophysiological measures in diverse settings^53^. The absence of substantiated evidence of reliable measurements in natural settings raises questions about their practical utility. Ensuring precision and consistency in psychophysiological measures is pivotal for robust conclusions regarding the link between exposure to natural settings and clinical research, necessitating rigorous testing and comprehensive validation of these instruments for generalizability in various ecological contexts^53,54^. Ambulatory assessment approaches come with unique challenges that might impact reliability. Physiological signals are obtained across a wide variety of environments and bodily states^55^, such that there is an inherent trade-off between reliability and validity^56^. Emphasizing reliability through standardized procedures and equipment settings can enhance consistency in measurements but may reduce validity by oversimplifying real-world phenomena. Conversely, improving ecological validity by sampling under a range of contextual settings undermines reliability^57–59^. In ambulatory assessment, the trade-off between reliability and validity becomes particularly evident under naturalistic conditions. When physiological measures are collected in real-world settings, factors such as weather, pedestrian traffic, noise levels, and participant movement introduce variability that can affect the consistency of the data^60^. For example, a participant’s physiological signals may fluctuate due to external conditions like temperature or traffic noise, which can compromise the reliability of the measurements. On the other hand, these naturalistic conditions enhance ecological validity by providing a more accurate reflection of how physiological responses occur in everyday life^5,53^. Balancing the need for standardized replicable protocols to ensure reliability with the imperative to maintain the ecological validity of measures is crucial for mitigating this trade-off.

This study aims to navigate this balance, recognizing that decisions made to enhance the reliability of psychophysiological measures in real-world contexts inevitably influence their validity, and thus, necessitate a thoughtful and context-specific approach. Nonetheless, despite the limited extent of reliability testing for psychophysiological parameters in natural settings, a deliberate and transparent attempt as illustrated in this study, is clearly warranted. To the best of our knowledge, this study represents the preliminary investigation into the test-retest reliability of multiple psychophysiological data within the context of navigating a real-world urban environment. Here we addressed this issue by examining the reliability of physiological measures (electrodermal- and cardiovascular activity) in healthy individuals when navigating an urban route under naturalistic conditions. Psychophysiological activity and outside temperature were recorded using mobile sensors on two separate testing days, and movement and location were tracked via global positioning system (GPS). The data were collected as part of a larger study investigating metabolic influences on psychophysiological responses, with participants tested once in a fasted state and once in a sated state. Short-term fluctuations in metabolic state may alter sympathetic-parasympathetic balance and hence psychophysiological responses. Individuals in a sated (baseline) state typically maintain homeostatic balance, with stable interactions between the SNS and the PNS^61^. Previous research reported inconsistent effects of short-term fasting on cardiovascular measures^62^. Herbert et al. (2012) reported an increase of heart rate and decrease of heart rate variability, consistent with increased SNS activity^63^, while others reported reduced heart rate and increased heart rate variability, suggesting increased PNS activity^64,65^. Together, these findings suggest that metabolic states can influence psychophysiological responses under some conditions. Since daily life, especially in urban environments, may involve frequent shifts in metabolic state due to various stressors or individual differences in food intake, this variability may impact the consistency of physiological measurements. The fact that data were obtained in two different metabolic states allowed us to examine whether hunger/satiety had a general impact on physiological measures, and whether the test-retest reliability of physiological measures is robust to such fluctuations in metabolic state. Overall, the test-retest reliability of a compound score across physiological measures (obtained via principal component analysis (PCA)) exceed that of most individual measure and was acceptable. Results show that even under highly uncontrolled naturalistic conditions, ambulatory physiological measures exhibit acceptable reliability.

## Methods

### Sample

Twenty-six healthy participants (*N* = 15 female, *Range*: 18–34 years) took part in the study. The sample size was selected based on practical and financial considerations (e.g. we had a time window of around 4 months for data acquisition). A post-hoc power analysis using G*Power 3.1.9.2^66^ (α = 0.05, power = 0.80) revealed that the study was sufficiently powered to detect a test-retest reliability of at least *r* =.449, corresponding to a medium effect size. Inclusion criteria included no history of cardiovascular, metabolic, gastrointestinal, psychiatric, or neurological disease, no medication or drug abuse. Participants were not on a diet and did not change their eating habits during the testing period. Vegetarians (*N* = 6) and vegans (*N* = 2) were included in the sample. Participants’ body mass index (BMI) was in the normal range (19.0 to 26.4 kg/m², *M* = 21.94, *SD* = 2.19), as was the percent body fat (*M*_female_ = 21.86, *SD* = 7.18; *M*_male_ = 14.72, *SD* = 4.57). All participants provided written informed consent and received a reimbursement of 10€ per hour for their participation. The study was approved by the ethics committee of the University of Cologne (code: KKHF0035) and conducted in accordance with the Declaration of Helsinki.

### General procedure

The data in this paper stem from a larger two-day study, assessing the effect metabolic state (sated vs. fasted condition) on various measures. Employing a counterbalanced within-subjects design, participants underwent two separate sessions on separate days, with a 6–8-day interval in between. On each testing day, participants walked the same urban route while undergoing an ambulatory psychophysiological assessment. The entire procedure was performed once in a sated state and once in a fasted state, in counterbalanced order. In the fasted state, participants were instructed to abstained from food intake for approximately 10 h before the testing session, while in the sated state, participants were instructed to consume their normal breakfast meal prior to the session.

**Fig. 1:**
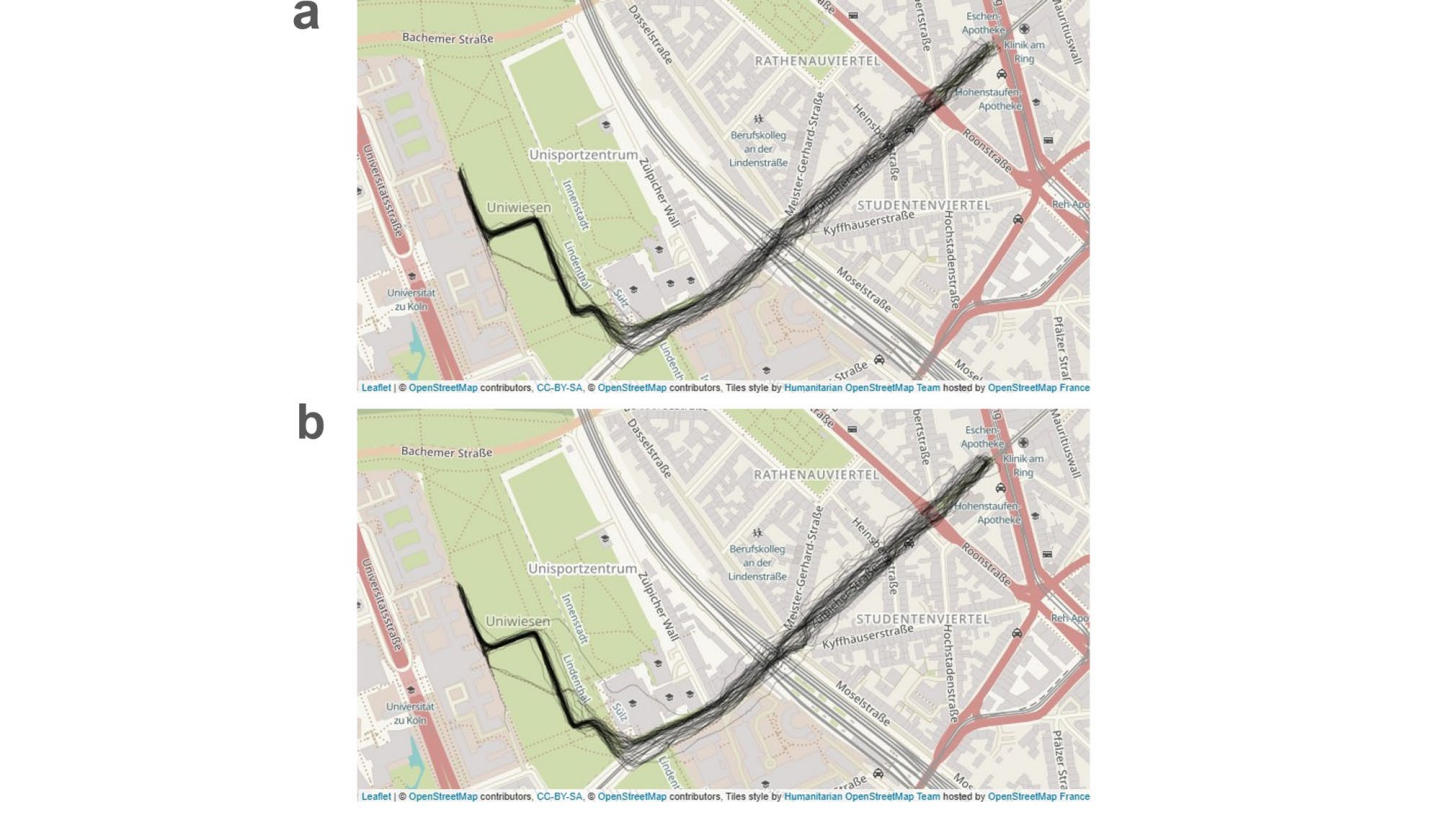
Urban walking route with single-subject trajectories. Urban walking route for day one (**a**) and day two (**b**). Individual-participant movement trajectories derived from GPS-based location tracking are depicted by black lines. Visualization created using Leaflet (https://leafletjs.com/), OpenStreetMap contributors^85^, CC-BY-SA (https://creativecommons.org/licenses/by-sa/2.0/), Tiles style by Humanitarian OpenStreetMap Team (https://www.hotosm.org/) hosted by OpenStreetMap France (https://www.openstreetmap.fr/) in R Studio 2024a^68^ with leaflet-package^86,87^.

On the first testing day, participants also underwent a baseline screening in the laboratory to check exclusion criteria. Additionally, information regarding dietary preferences such as veganism or lactose intolerance was collected. A Bioelectrical Impedance Analysis (body fat percentage) and the BMI were performed for each participant, followed by the completion of several questionnaires, including socio-demographic details and subjective assessment of tiredness and hunger levels. Participants were then provided with a detailed map of the urban walking route and were instructed to memorize it after a thorough explanation. The route included three intersections with traffic lights, a small path between two green areas and an avenue of trees leading to and from the main street (see Fig. 1). Subsequently, mobile electrocardiograph (ECG) and EDA sensors were applied (see next section). A study smartphone equipped with GPS recording capabilities was provided to the participant. Communication with other people or listening to music was prohibited during the urban walking exposure. Participants were instructed to walk at a constant, previously trained speed (*M* = 4.1 km/h, *SD* = 0.27). The urban route was 2.1 km in length and took participants approximately 30 min to complete. Upon return, GPS recording was terminated, and electrodes were removed from the participant in the laboratory. Participants were asked about any noteworthy events they may have experienced during the urban walking route and the experimenter noted them accordingly. After route completion participants completed an additional laboratory task, to assesses subjective evaluation of food rewards. Testing was conducted in the morning, between 8 AM and 12 PM, with each participant walking both routes at a similar time within this period, although the specific time for each participant varied.

### Ambulatory assessment

#### Physiological recording

To measure ANS activity, electrodermal and cardiovascular data were recorded using the mobile ECGmove4 and EDAmove4 devices from Movisens^67^. Both devices store data in terms of the system time of the associated computer they were coupled with, ensuring synchronization of time stamps across channels. The ECGmove4 was placed under the left chest of the participant using disposable adhesive pre-gelled Ag/AgCI electrodes and acquired raw data (sampling rate 1024 Hz) of a single channel ECG, from which secondary parameters like heart rate variability (ms) and heart rate (bpm) were calculated. The EDAmove4 collected skin conductance level (µS), skin conductance response (count), and skin conductance response-amplitude (µS) data to reflect EDA measures. The EDA device was fixed to a strap band on the non-dominant wrist. The EDA pre-gelled disposable adhesive Ag/AgCI electrodes were attached to the thenar and hypothenar eminences of the non-dominant palm. Further, the sensor applied a DC voltage of 0.5 V to the skin to gain skin conductance with a 32 Hz sampling rate.

#### GPS-based location tracking

Participants were equipped with a study smartphone for continuous GPS-location tracking. Time and date on the GPS location tracking app (GPS Logger version 2.1.12) were synchronized with the mobile physiological sensors via the linked computer. The tracking of the instantaneous position was performed at an interval of one second to ensure high accuracy.

### Data analysis

#### Physiological data

Statistical analyses were carried out using RStudio ^68^ and Matlab 2019a (MathWorks) statistical software^69^. Physiological data were processed using the custom Movisense “DataAnalyzer”-software and using custom Matlab code. Generally signal quality was assessed via visual inspection, preprocessing, correction for artifacts from motion, and involved an alignment of recordings to the 30-minute urban walking route. For EDA, preprocessing was performed by the Movisens software, which applies a built-in low-pass filter to remove high-frequency noise. The software’s detection parameters are based on established standards in psychophysiological research, specifically Dawson et al. (2000) and Boucsein (2012), ensuring consistency with validated EDA analysis^13,70^. ECG data were acquired at a sampling rate of 1024 Hz and preprocessing was also performed by the Movisens software. R-peak detection followed an adapted version of the algorithm by Hamilton (2002)^71^. Post-processing involved filtering R-peaks using a method adapted from Clifford et al. (2002)^72^. Due to missing values in heart rate variability indices from the manufacturer’s output, we used a custom Matlab script to extract R-peaks directly from the raw ECG signals, enabling heart rate variability analysis. This was necessary to allow for heart rate variability analysis in synchronization with GPS data. To prepare the signal for detection, ECG traces were first z-standardized. R-peaks were then identified using Matlab’s findpeaks function, applying a minimum peak height of one standard deviation and a minimum peak distance of 500 ms to exclude noise and physiologically implausible detections. Detection accuracy was ensured by cross-validating against R-peaks identified by the Movisens software. Comparison showed high consistency in peak timing. Subsequently, the variance between R-R intervals was computed as the RMSSD, a standard time-domain heart rate variability metric^45,73^.

#### Data processing

For each participant and physiological parameter of interest (heart rate, skin conductance level, skin conductance response amplitude, and skin conductance response count), physiological measures were computed in successive sixty second time bins, covering the entire 30-minute route. Furthermore, physiological measures were segmented into five recording epochs, including baseline-urban green space (park), three segments of urban grey space (exposure to shops and restaurants), and post-urban green space (park). To achieve this, GPS data were co-registered with the mobile sensors time stamps, ensuring consistent temporal alignment. The route with predefined start and end coordinates (latitude: 50.9272495, longitude: 6.9343163) served as a reference for epoch delineation. Participants movement along this route was tracked, with the initiation of the urban grey space phase marked by proximity to these coordinates and its conclusions indicated by deviation from the specified route. Subsequently, the urban grey space was subdivided into three equal segments. Following segmentation, physiological measures were averaged per segment, and z-standardized within-participants, separately for each testing day. In addition, in analyzing the comprehensive physiological responses along the entire walking route, the physiological measures were averaged across all epochs for each participant and each testing day, covering the entirety of the walking route. We subdivided the physiological measures collected during the study into two distinct partitions. The first partition segmented data into five segments. The second partitioning used the average physiological data across the entire recording epoch for each participant and session, providing a comprehensive overview of physiological responses throughout the entire walking route. These partitions offer complementary perspectives on the temporal dynamics of physiological activity during the study.

#### Principal component analysis

To account for covariance between physiological measures, we applied PCA. PCA is a multivariate statistical technique for data-driven dimensionality reduction that identifies directions of shared variance in high-dimensional data. PCA identifies linear combinations of the original variables that project the data onto orthogonal axes. Principal components across measures may constitute more robust physiological indices, compared to individual physiological measures^74^. Given the complexity of physiological data, which often includes several correlated variables, PCA helps to reduce this complexity by summarizing the data into a smaller number of principal components^75^. This reduction simplifies the comparison of physiological patterns across different testing sessions by focusing on these key components rather than the original, potentially noisier, measures. By extracting the principal components, PCA effectively filters out noise and emphasizes the most significant patterns, which is crucial for assessing how consistently physiological responses are replicated over time.

Based on two distinct partitioning’s derived from the physiological measurements (as described above), PCA was conducted separately for each partitioning. For the first partition, separate PCAs were computed for each segment of the data, per day. In contrast, for the partition covering the entire walking route, a single PCA was computed across all physiological measure, separately for each day. This approach captured variations in physiological responses over both testing days, facilitating exploration of underlying patterns. By integrating PCA into our analysis, we effectively managed the complexity of the data and focused on the most relevant patterns of variation, ensuring a robust assessment of reliability^74^.

#### Reliability assessment

Test-retest reliability of principal component scores and individual physiological measures were computed using a bootstrapping approach. Bootstrapping involves creating multiple simulated samples from the original data by resampling data points with replacement. This method provides a robust and flexible estimate of reliability, accommodating non-normality and outliers in the data, and ensures results are not overly influenced by the specific sample^76^. For each measure, 10,000 bootstrap samples were created via resampling data points at the subject level, and the Spearman (*r*_S_) correlation coefficient (correlation between day one and day two measures) was computed for each bootstrap sample. The median of the correlation coefficient distribution across samples was taken as a summary measure. The reliability assessments followed established criteria, recognizing that reliability estimates are continuous measures^77^. Rather than relying on arbitrary cutoffs, some researchers suggest labeling intervals of reliability^78^. Reliabilities were classified as follows: *r*$\:\:\le\:$ 0.30: low; 0.31$\:\le\:$ *r* $\:\le\:$ 0.50: moderate; 0.51$\:\le\:$ *r* $\:\le\:$ 0.70: acceptable^79^, 0.71$\:\le\:$ *r* $\:\le\:$ 0.80: good^80^, 0.81$\:\le\:$ *r* $\:\le\:$ 0.90: very good^81^, *r* >.90: excellent^82^. Note that such labels provide additional context for interpreting reliability estimates, and should not be viewed as strict thresholds^83^.

A split-half reliability analysis was performed separately for each day using the preprocessed segmented data (5 epochs), extracting the first principal component of each epoch. This method is employed to assess internal consistency, ensuring that different parts of the data yield consistent results. To divide the data, we adopted an even-numbered approach, comparing epochs 1 and 2 against epochs 4 and 5 for each day separately. This approach enhances the estimation of internal consistency by examining distinct temporal segments within the data. Epoch 3 was excluded from the split to maintain a balanced comparison between the two halves. The use of split-half reliability provides a robust measure of the stability and reliability of the data across time^84^.

## Results

GPS tracking confirmed that participants consistently adhered to the prescribed walking route (see Fig. 1a and b for individual participant movement trajectories). On day 1, the average distance across participants was *M* = 2.23 km (± SD = 0.11) and the average walking duration was *M* = 30.94 min (± SD = 1.51). On day 2, the average distance across participants was *M* = 2.24 km (± SD = 0.10) and the average walking duration was was *M* = 30.58 min (± SD = 1.14). Throughout the study, environmental temperatures ranged between 16° and 21 ° degrees Celsius (*M* = 18.34°, ±SD = 1.19). On day 1 average temperature was *M* = 18.26° degree Celsius (± SD = 1.28, *Range* = 16.2°−21° degrees Celsius) and on day 2 *M* = 18.42° degree Celsius (± SD = 1.06, *Range* = 16.5°−20.3° degrees Celsius).

**Fig. 2:**
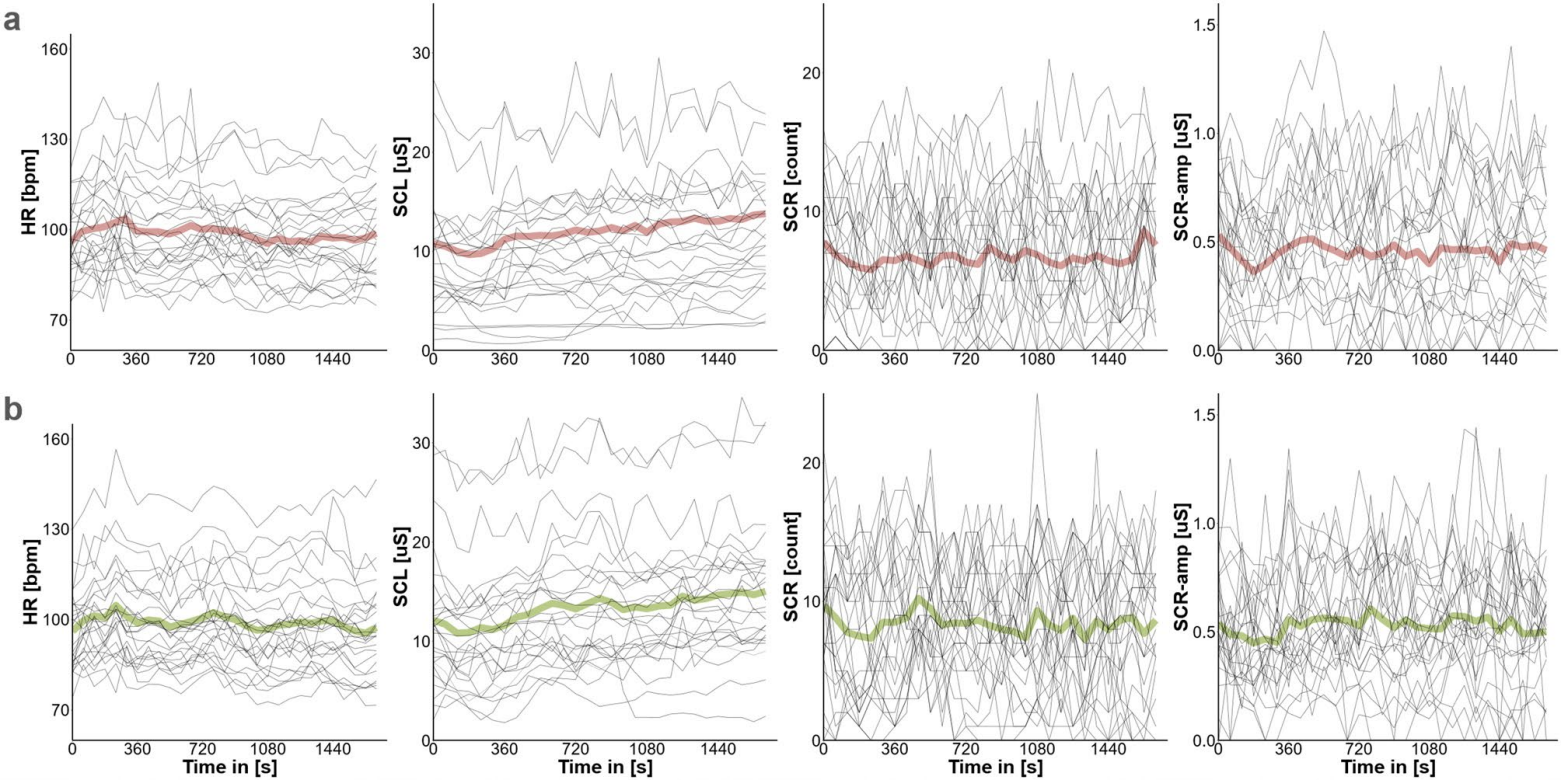
Physiological measures for single individual participants (thin lines, *N* = 26) and group averages (thick lines) across time for day one (**a**) and day two (**b**). HR = Heart Rate, SCL = Skin Conductance Level, SCR-amp = Skin Conductance Response- Amplitude, SCR = Skin Conductance Response count.

As a first step, we visualized trajectories of individual mean physiological measurements across the route (averaged for bins of sixty seconds). On both testing days (see Fig. 2), the physiological measures observed in our study fell within the plausible ranges, with considerable variability across participants. Resting heart rate typically ranges between 60 and 100 bpm depending on age and fitness level, and can increase to approximately 90–120 bpm during light to moderate physical activity such as walking^88,89^. Skin conductance levels are generally reported between 1 and 40 µS, while skin conductance response amplitudes typically range from 0.5 to 2 µS for low-amplitude responses, with higher values (up to 10 µS) associated with strong emotional or physiological arousal^13^. The frequency of skin conductance response counts under resting conditions is typically around 3–5 responses per minute but may rise to 10–15 per minute in highly arousing situations^13,14^. Our measures were consistent with these ranges, considering within-subject variability.

**Fig. 3:**
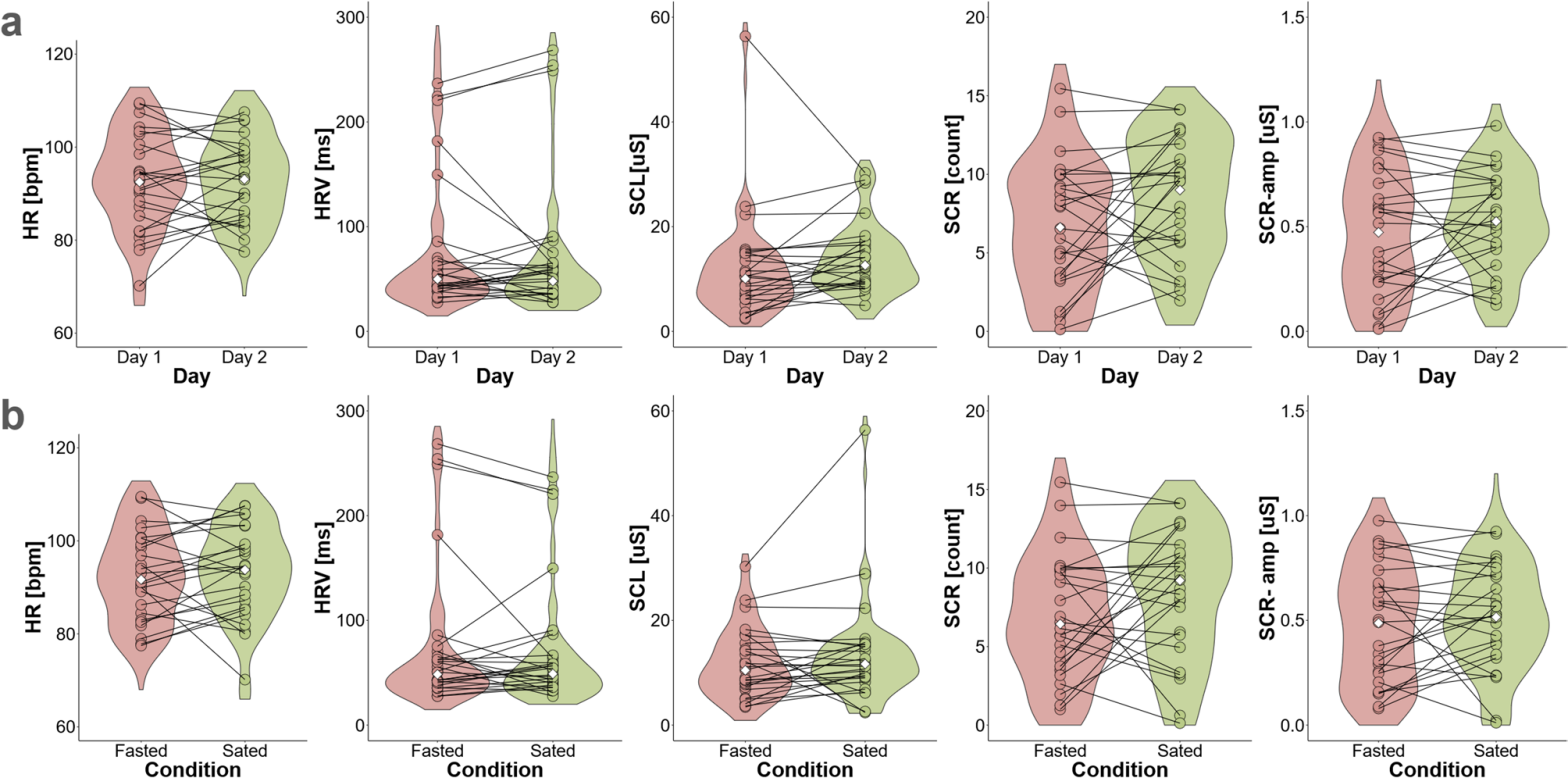
Distributions of mean physiological measures across days and conditions. Violin plots depicting mean physiological data from both testing days (panel **a**) and from both conditions (panel **b**); dots represent single-subject means of each day/condition; white diamond mark = median. HR = Heart Rate, HRV = Heart Rate Variability, SCL = Skin Conductance Level, SCR-amp = Skin Conductance Response-Amplitude, SCR = Skin Conductance Response count.

Next, we separated data according to testing day (day one vs. day two, see Fig. 3a) and condition (sated vs. fasted, see Fig. 3b), to explore potential order and/or metabolic modulation effects. To account for potential order effects, we also present the data separately for day one and day two, as initial exposure to the experimental protocol may have influenced physiological responses.

Differences in single physiological measures between testing days (day one vs. day two) and conditions (fasted vs. sated) were assessed via Wilcoxon signed rank tests adjusted for multiple comparisons using Bonferroni correction. None of the comparisons were significant (see Table 1). As expected, there was considerable covariance in physiological measures, and the overall pattern was highly consistent across day one (Table 2) and day two (Table 3) for Pearson bivariate correlations of single physiological measures and temperature. None of the physiological measures showed significant associations with environment temperature.

**Table 1:** Wilcoxon signed-rank test of single physiological measures across conditions and testing days bonferroni corrected. *N* = 26. HR = Heart Rate, HRV = Heart Rate Variability, SCL = Skin Conductance Level, SCR-amp = Skin Conductance Response- Amplitude, SCR = Skin Conductance Response count.

	Condition					Day				
	Fasted		Sated			Day 1		Day 2		
	*M*	*SD*	*M*	*SD*	*p*	*M*	*SD*	*M*	*SD*	*p*
HR	90.37	12.24	93.17	9.59	0.65	92.44	10.29	91.55	11.25	> 0.999
HRV	79.25	71.98	72.72	56.47	> 0.999	78.18	64.83	75.80	68.90	> 0.999
SCL	11.93	6.71	13.61	10.38	0.47	12.11	10.58	14.26	6.84	0.12
SCR	6.91	3.86	8.33	3.97	> 0.999	6.79	4.07	8.46	3.70	0.65
SCR-amp	0.50	0.25	0.51	0.24	> 0.999	0.46	0.29	0.53	0.23	0.47

**Table 2:** Pearson bivariate correlation matrix of single physiological measures and temperature on day one. Adjustment for multiple comparisons: bonferroni. *N* = 26, correlation significant at *p >.05. HR = Heart Rate, HRV = Heart Rate Variability, SCL = Skin Conductance Level, SCR-amp = Skin Conductance Response- Amplitude, SCR = Skin Conductance Response count.

Variables	HR	HRV	SCL	SCR-amp	SCR	Temp
HR	−	− 0.222	0.263	0.289	0.281	− 0.250
HRV		−	− 0.032*	0.175	0.226	0.197
SCL			−	0.715*	0.195	− 0.186
SCR-amp				−	0.634	0.046
SCR					−	0.102
Temp						−

**Table 3:** Pearson bivariate correlation matrix of single physiological measures and temperature on day two. Adjustment for multiple comparisons: bonferroni. *N* = 26, correlation significant at *p >.05. HR = Heart Rate, HRV = Heart Rate Variability, SCL = Skin Conductance Level, SCR-amp = Skin Conductance Response- Amplitude, SCR = Skin Conductance Response count.

Variables	HR	HRV	SCL	SCR-amp	SCR	Temp
HR	−	− 0.508	− 0.156	0.055	− 0.044	− 0.177
HRV		−	0.007	− 0.332	− 0.255	0.100
SCL			−	0.626*	0.217	0.412
SCR-amp				−	0.358	0.360
SCR					−	0.378
Temp						−

### Principal component analysis

**Fig. 4:**
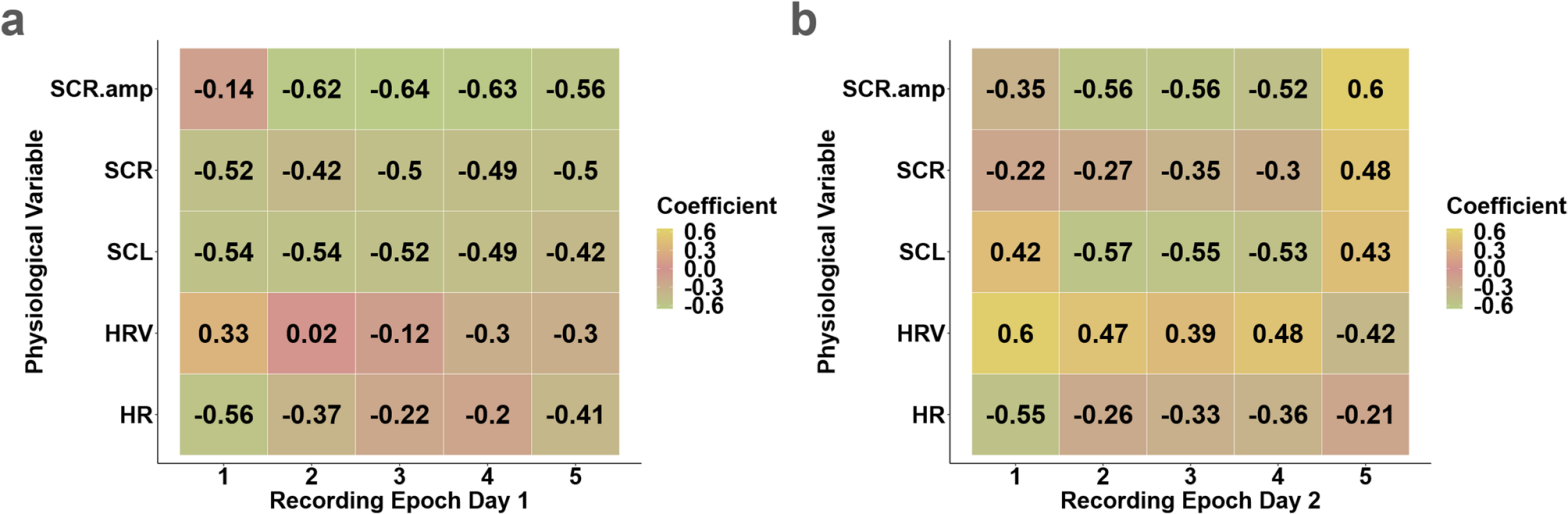
Heatmap of the loadings (coefficients) of the PC#1 from a PCA of single physiological measures, shown across recording epochs. Loadings of PC#1 are shown for day one (**a**) and day two (**b**). HR = Heart Rate, HRV = Heart Rate Variability, SCL = Skin Conductance Level, SCR-amp = Skin Conductance Response- Amplitude, SCR = Skin Conductance Response count.

First, the data were split into five recording epochs per day (see methods section). For each epoch, a PCA was computed across physiological measures, separately for day one (Fig. 4a) and day two (Fig. 4b). Coefficients of the first principal component (PC#1) were mostly consistent across the recording epochs for both days (see Fig. 4). For example, heart rate and EDA measures exhibited predominantly negative loadings on the PC#1.

A split-half reliability analysis was performed to assess the internal consistency of the physiological variables across epochs for each day. Epoch 3 was excluded to ensure balanced comparisons, with aggregated PC#1 scores from epochs 1 and 2 compared to scores from epochs 4 and 5. On day 1, the bootstrapped Spearman correlation coefficient was *r*_S_ =0.85, indicating good reliability, whereas day 2 showed low reliability (*r*_S_ =0.31), driven by a strong negative correlation between epochs 4 and 5 (*r*_S_ =−0.72), specifically in PC#1 in epoch 5. While PCA is sign-invariant, such flips introduce arbitrary variability^75^, affecting Spearman correlations, which are sensitive to directionality. The low reliability observed on day 2 was possibly not due to a change in physiological state, but rather may have resulted from polarity shifts in the principal components and the sensitivity of the Spearman correlation to directionality. To address this, reliability was reassessed by comparing aggregated scores from epochs 1 and 2 to those from epochs 3 and 4. This yielded improved reliability estimates *r*_S_=0.87 for day 1 and *r*_S_ =0.91 for day 2. Including epoch 3 allowed for a more balanced and consistent comparison, removing the influence of polarity shifts and providing more reliable estimates of temporal consistency within days.

Additionally, to comprehensively examine physiological responses throughout the walking route, the mean values of physiological measures were computed for each participant and testing day across all epochs. For each testing day a PCA across all physiological measures and participants was calculated. The PC#1 accounted for 39–45% of the variance across physiological measures on two distinct days (see Table 4). PC#1 coefficients were again highly consistent across recording days (see Table 5). This implies that the overall covariance pattern across physiological measures was stable across testing days.

**Table 4:** Contribution of the variance explained by all components, derived from the individual physiological measurements on different days across all epochs. HR = Heart Rate, HRV = Heart Rate Variability, SCL = Skin Conductance Level, SCR-amp = Skin Conductance Response- Amplitude, SCR = Skin Conductance Response count.

	Day 1			Day 2		
Component	Eigenvalue	% of variance	Cumulative %	Eigenvalue	% of variance	Cumulative %
1	2.26	45.10	45.10	1.96	39.27	39.27
2	1.24	24.87	69.98	1.50	30.11	69.39
3	0.84	16.71	86.70	0.82	16.46	85.85
4	0.53	10.61	97.38	0.42	8.46	94.31
5	0.13	2.61	100.00	0.28	5.69	100.00

**Table 5:** Coefficients of single physiological measures on different days across all epochs. Loadings of the first three principal components (PC#1, PC#2, PC#3) of the single physiological measurements at different days. HR = Heart Rate, HRV = Heart Rate Variability, SCL = Skin Conductance Level, SCR-amp = Skin Conductance Response- Amplitude, SCR = Skin Conductance Response count.

	Day 1			Day 2		
Variable	PC#1	PC#2	PC#3	PC#1	PC#2	PC#3
HR	− 0.34	0.51	0.53	− 0.15	0.69	0.29
HRV	− 0.10	− 0.79	0.05	0.40	− 0.56	0.07
SCL	− 0.50	0.18	− 0.65	− 0.48	− 0.44	0.42
SCR-amp	− 0.49	− 0.25	0.53	− 0.44	− 0.07	− 0.83
SCR	− 0.62	− 0.10	− 0.18	− 0.62	− 0.15	0.24

**Fig. 5:**
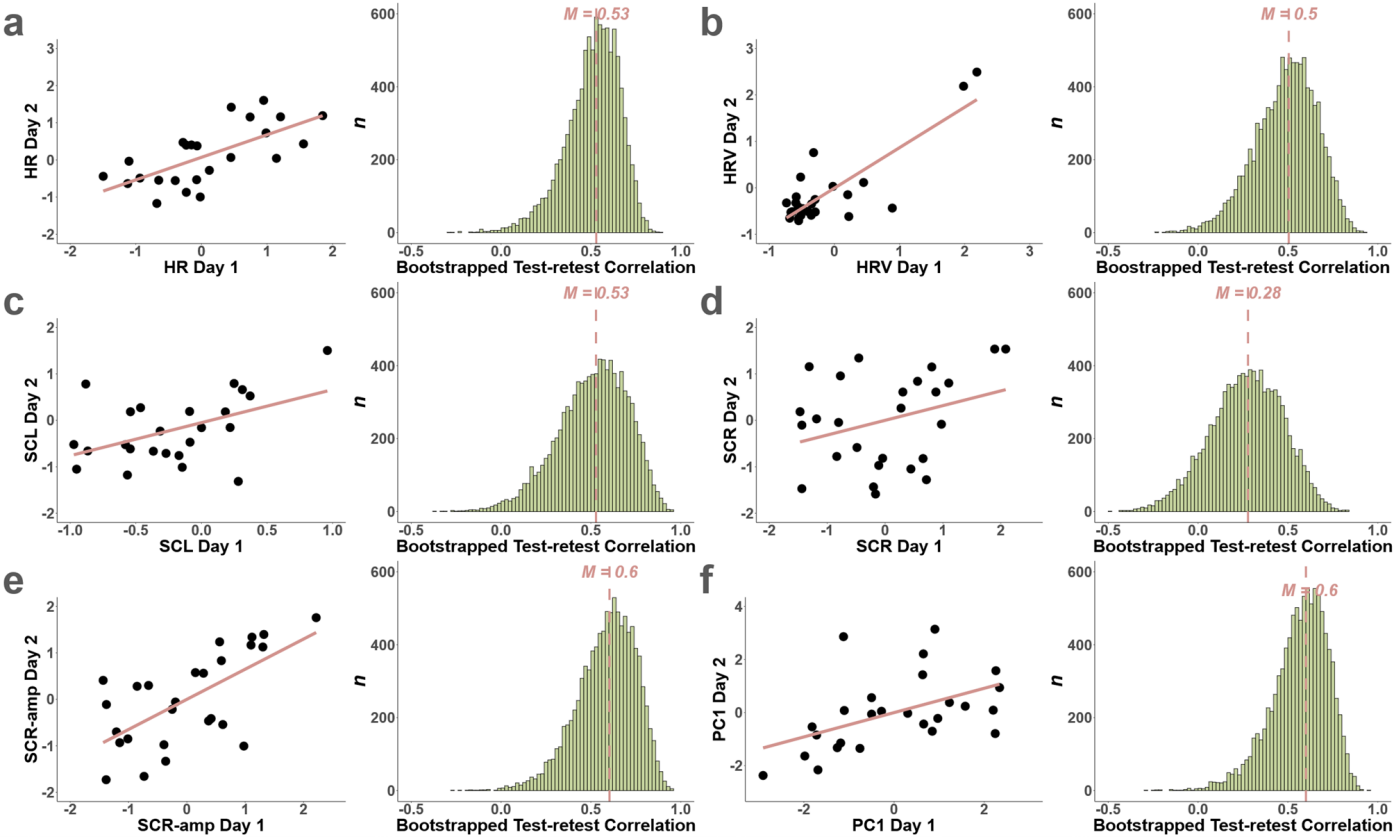
Bootstrapped test-retest analyses of single physiological data. Bootstrapped test-retest reliability of single physiological measures (**a**−**e**) over day 1 and day 2 (panel **a**) and bootstrapped test-retest correlation across 10k bootstrap samples (panel **b**; dashed red line: median correlation coefficient across samples. HR = Heart Rate, HRV = Heart Rate Variability, SCL = Skin Conductance Level, SCR-amp = Skin Conductance Response- Amplitude, SCR = Skin Conductance Response count.

We next used a bootstrapping approach to estimate the test-retest reliability of each individual physiological measure as well as for the compound score of PC#1, across testing days (see Fig. 5a-f). The median Spearman correlation coefficients across bootstrap samples indicate a low to acceptable reliability for individual physiological measures (HR: *r*_S_ = 0.53, HRV: *r*_S_ = 0.50, SCL: *r*_S_ = 0.53, SCR: *r*_S_ = 0.28, SCR-amplitude: *r*_S_ = 0.60). The reliability of PC#1 was acceptable (*r*_S_ = 0.60; see Fig. 5f).

## Discussion

In a counterbalanced repeated measures within-subjects design, we examined the test-retest reliability of ambulatory psychophysiological measures obtained under naturalistic ambulatory assessment conditions. Cardiovascular and electrodermal activity were continuously recorded using mobile sensors, in combination with GPS-based location tracking, while participants walked the same urban route on each testing day. The first principal component across physiological measures accounted for between 39% and 45% of variance across physiological measures. PC#1 scores from the aggregated measure exhibited acceptable test-retest reliability across testing days, suggesting that compound scores across physiological measures may provide reliable physiological indices under naturalistic conditions.

The loading pattern of PC#1 suggests that it captures the balance between parasympathetic and sympathetic tone, with highly similar patterns on both testing days. The negative association with most physiological measures suggests that higher scores on this component are indicative of reduced sympathetic tone. This interpretation is supported by the lower loadings observed for heart rate and EDA-based measures. Heart rate and heart rate variability tend to be inversely correlated^17,45,90^. The loading pattern observed for day two was consistent with this finding (i.e. PC#1 exhibited negative loadings for heart rate and positive loadings for heart rate variability). For testing day one, this pattern was less pronounced, and here heart rate variability showed a numerically small negative loading for PC#1. Nonetheless, the overall loading pattern for PC#1 was similar across testing days, and PC#1 score exhibited acceptable test-retest reliability across testing days.

In contrast, individual physiological measures demonstrated more variability in test-retest reliabilities, spanning a range from low to acceptable reliabilities. In particular the skin conductance response count measure exhibited low reliability. EDA measurements are especially susceptible to environmental fluctuations, movement artifacts^91^, outside temperature and humidity^13^. Such effects may influence EDA measurements more than cardiovascular measures, in particular during ambulatory recordings. However, we did not observe associations of EDA measures with outside temperature, suggesting that this factor did not play a central role in the present study. The skin conductance level exhibited considerable variation among participants, ranging from minimal responses to increased conductance levels, consistent with the idea that this measure varies greatly inter-individually^14,91^. Similarly, ECG-derived variables, such as heart rate variability, showed some outliers. This variability in some of the physiological measures highlights the utility of pooling information across them. Findings from the composite score (i.e. PC#1) indicate acceptable results in assessing participants’ physiological responses under naturalistic conditions, confirming the usefulness of combining information across multiple physiological measures in order to improve reliability.

The split-half reliability analysis demonstrated a consistent pattern of internal consistency across epochs and days, except on day 2, epoch 5. On day 1, reliability remained high when comparing aggregated PC#1 scores from epochs 1 and 2 with either epochs 3 and 4 or epochs 4 and 5, indicating strong consistency in psychophysiological measures. However, day 2 showed low reliability when comparing epochs 1 and 2 with epochs 4 and 5, due to a sign flip in the PC#1 in epoch 5. Although PCA is sign-invariant—preserving the structure and variance it captures regardless of direction^92,93^—such sign flips introduce arbitrary variability into subsequent analyses^75^. This variability, unrelated to the underlying data, affects Spearman correlation coefficients, which are sensitive to the directionality of the PC#1 loadings^94^. To address this issue, we adjusted the split-half comparison for day 1 and day 2 to use epochs 3 and 4 as the second half, which resulted in high reliability estimates. These findings emphasize, despite the isolated issue in epoch 5, that the overall results demonstrate good internal consistency across temporal segments on each day.

Comparing the reliability estimates of physiological data obtained in controlled laboratory settings with those acquired under real-world, naturalistic conditions offers valuable insights into the robustness of physiological measures in diverse environments. Our findings indicate noteworthy similarities between the reliability estimates obtained in laboratory settings and those observed in naturalistic conditions. Specifically, cardiovascular indices such as heart rate and heart rate variability exhibit comparable moderate reliability estimates across both settings^43,45^. For instance, Mathar et al. (2020) reported test-retest reliabilities (intraclass correlation coefficient) of 0.74 for heart rate and 0.78 for heart rate variability under laboratory conditions, showing a trend consistent with our findings of 0.53 and 0.50, respectively, under natural conditions. While our estimates exhibit a marginally lower reliability compared to those obtained in the laboratory, they nonetheless demonstrate a robust consistency. As such, the present results underscore a general robustness of cardiovascular indices across diverse environmental contexts and their efficacy as indicators of autonomic nervous system activity. Moreover, our investigation delved into the reliability of EDA measures, which are sensitive indices of sympathetic nervous system activation^13,95^. Our results reveal that EDA measures maintain low to acceptable reliability levels during urban mobility. Notably, both skin conductance level and skin conductance response-amplitude demonstrate acceptable reliability estimates. Similar, laboratory studies show moderate reliability estimates of EDA measures^46–48^. While laboratory settings offer controlled conditions for data collection, our results suggest that physiological measures remain robust and reliable even in the face of environmental variability encountered in naturalistic settings. Moreover, our study highlights the potential advantages of employing a compound score derived from multiple physiological variables as a more comprehensive approach. By integrating data from various physiological measures, including cardiovascular and electrodermal indices, the compound score provides a summary representation of physiological response. This approach offers a more robust and nuanced understanding of participants’ reactions under naturalistic conditions, surpassing the limitations of single-variable analysis commonly employed in traditional lab-based research^5,96^.

This study further examined whether metabolic state (fasted vs. sated) influences physiological responses during urban mobility. Contrary to expectations, no significant differences were found, indicating that metabolic state did not affect physiological reactions. The body’s ability to maintain homeostasis likely explains the lack of significant physiological differences between fasted and sated states. This regulatory capability ensures that internal conditions remain stable despite external environmental changes encountered during urban mobility^97^. The physiological responses measured in studies might be more strongly influenced by other urban stimuli, including noise, traffic, and crowd density, which can have pronounced impacts on the body’s stress responses. For instance, studies have demonstrated that urban noise can significantly elevate cortisol levels and increase heart rate, indicating heightened stress^98^. Urban dwellers are frequently exposed to a multitude of stimuli, which can lead to sensory adaptation and reduced sensitivity^99^. This desensitization might help explain why metabolic state-specific responses are less pronounced in such environments. Furthermore, our results demonstrate that even though testing occurred in different metabolic states, the test-retest reliability of the physiological measures was still acceptable. This is an important aspect for future studies collecting real-world physiological data, as results suggest that short-term fluctuations in metabolic state may only have a limited impact on the reliability of physiological outcomes.

We focused on reliability of psychophysiological measures during navigation in a naturalistic real-world environment. However, under such conditions, various factors might influence state-dependent changes in physiological measures. For instance, positive mood states are associated with an increased vagal influence on heart rate^100^. Baseline measurements and a GPS-based control of movement speed, as done in the present study, can help mitigate some of these effects. However, a 30-minute walk in a natural setting introduces the potential for unexpected stressors and/or mood fluctuations. Not all variations in mood and stressors can be fully accounted for, as participants may encounter unanticipated complexities during route exposure^53,60^. While most participants did not report any incidents, some individuals reported specific experiences, such as encountering sirens or friends, or navigating dilemmas, all of which may potentially affect physiological responses. Such unsystematic effects likely lead to lower reliability estimates as compared to laboratory-based studies. However, put differently, despite the potential presence of uncontrolled factors, reliability was overall acceptable, in particular for the PCA-based compound score. By demonstrating the feasibility of obtaining reliable physiological data in real-world contexts, our study enhances the applicability and generalizability of physiological research, facilitating more ecologically valid assessments of human physiology. As this is a prerequisite for applications in (clinical) psychology, wearable health technologies, and digital health, the present results provide preliminary support for such future employment. Further research is, however, needed to delve into the specific factors driving potential patterns and their implications for human health and well-being.

### Limitations and future directions

As this study explored the reliability of physiological responses in a dynamic urban environment, several limitations emerged. First, we focused on the reliability of physiological measures across two testing days with a test-retest interval of around one week. Future research may delve deeper into the long-term stability of physiological measures. Second, the influence of external factors on physiological measures, such as environmental conditions (with the exception of temperature) were not controlled, such that the reliability estimates provided here constitute a lower bound. In this context, single-subject GPS trajectories revealed minor deviations from the intended experimental route, more tightly controlled experimental designs or recording conditions might yield higher estimates. Third, individual responses to specific stressors or events might reflect individual differences more than continuous physiological recordings such as carried out here. Future studies are required to explore this possibility. Fourth, we did not obtain subjective measures (e.g. stress or arousal ratings), such that the association between physiological and subjective measures remains unclear. A further limitation is that the present study focused on physiological measures. Future work should additionally obtain subjective measures to further explore the links between subjective and objective indices related to urban environments. Lastly, ambulatory monitoring studies are often done in relatively small samples due to the complexity of data acquisition, and this was also the case here. Future studies will benefit from larger samples sizes and a-priori (rather than post-hoc) power calculations. In addition, ambulatory measurements conducted in real-world environments are inherently subject to several challenges, including potential variability due to movement and environmental factors that cannot be fully controlled. Consequently, heart rate variability could not be reliably visualized over time due to missing data and signal artifacts, which limits temporal interpretation. Moreover, decisions made during data processing and analysis, although based on best practices, may introduce biases or uncertainties that could influence the results. These limitations should be carefully considered when interpreting the findings, and future research would benefit from further methodological refinements aimed at improving the reliability, validity, and generalizability of data collected during ambulatory assessments. Future studies should replicate and test the reliability of psychophysiological data obtained in urban environments and/or under naturalistic conditions, and account for additional environmental effects and/or individual differences in response profiles. Future research might also further explore the associations of self-report measures and physiological effects.

## Conclusion

We examined the reliability of physiological measures obtained during naturalistic urban mobility conditions. Reliability of physiological measures is of central importance for studies that aim to shed light on how individuals navigate and respond to the complex interplay of environmental factors in natural settings. Our findings demonstrate an acceptable reliability of a PCA-based compound score across cardiovascular and electrodermal measures, which largely represented sympathetic tone during urban mobility. This confirms that physiological reactivity can reliably be estimated in a real-world urban setting, an important prerequisite for future applications of ambulatory psychophysiological assessments under real-world conditions. Contextual and environmental effects on physiological responses hold significant potential for ecologically valid future research applications in applied and clinical contexts in psychology and clinical neuroscience.

## Data Availability

The data that support the findings of this study are openly available at the Open Science Framework (https://osf.io/fesvm/?view_only=b2cf851c78ef4867a5b4231fc36a0fc8).

## References

[1] Van Roekel, E., Keijsers, L. & Chung, J. M. A review of current ambulatory assessment studies in adolescent samples and practical recommendations. *J. Res. Adolescence*. **29**, 560–577 (2019). 10.1111/jora.12471 PMC6790669 31573762

[2] Ebner-Priemer, U. W. & Trull, T. J. Ambulatory assessment: an innovative and promising approach for clinical psychology. *Eur. Psychol.* **14**, 109–119 (2009).

[3] Carpenter, R. W., Wycoff, A. M. & Trull, T. J. Ambulatory assessment: new adventures in characterizing dynamic processes. *Assessment* **23**, 414–424 (2016). 26887808 10.1177/1073191116632341 PMC6410721

[4] Reichert, M. et al. Ambulatory assessment for physical activity research: state of the science, best practices and future directions. *Psychol. Sport Exerc.* **50**, 101742 (2020). 32831643 10.1016/j.psychsport.2020.101742 PMC7430559

[5] Trull, T. J. & Ebner-Priemer, U. Ambulatory assessment. *Ann. Rev. Clin. Psychol.* **9**, 151–176 (2013). 23157450 10.1146/annurev-clinpsy-050212-185510 PMC4249763

[6] Orne, M. T. On the social psychology of the psychological experiment: with particular reference to demand characteristics and their implications. *Am. Psychol.* **17**, 776–783 (1962).

[7] Orr, S. P. & Roth, W. T. Psychophysiological assessment: clinical applications for PTSD. *J. Affect. Disord.* **61**, 225–240 (2000). 11163424 10.1016/s0165-0327(00)00340-2

[8] Kohen, C. B., Cofresí, R. U., Bartholow, B. D. & Piasecki, T. M. Alcohol craving in the natural environment: moderating roles of cue exposure, drinking, and alcohol sensitivity. *Exp. Clin. Psychopharmacol.* **31**, 57–71 (2023). 35025586 10.1037/pha0000540 PMC9276840

[9] Fisher, J. P., Young, C. N. & Fadel, P. J. Autonomic Adjustments to Exercise in Humans. In: Prakash YS (ed) Comprehensive Physiology, 1st ed. Wiley, pp 475–512 (2015). 10.1002/cphy.c140022 25880502

[10] Gibbons, C. H. Basics of autonomic nervous system function. In K. H. Levin, & P. Chauvel (Eds.), *Handbook of Clinical Neurology* (pp. 407–418). Elsevier. 10.1016/b978-0-444-64032-1.00027-8 (2019). 10.1016/B978-0-444-64032-1.00027-8 31277865

[11] McCorry L.K. Physiology of the autonomic nervous system. *Am J Pharm Educ*. **71**(4), 78. 10.5688/aj710478 (2007). 10.5688/aj710478 PMC1959222 17786266

[12] Wehrwein, E. A., Orer, H. S. & Barman, S. M. Overview of the anatomy, physiology, and Pharmacology of the autonomic nervous system. In: (ed Terjung, R.) Comprehensive Physiology, 1st ed. Wiley, 1239–1278 (2016). 10.1002/cphy.c150037 27347892

[13] Boucsein, W. *Electrodermal Activity* 2nd edn (Springer Science & Business Media, 2012).

[14] Braithwaite, J., Watson, D. G. & Jones, R. A guide for analysing electrodermal activity (EDA) & skin conductance responses (SCRs) for psychological experiments. 1017–1034 (2013).

[15] Chapleau, M. W. & Sabharwal, R. Methods of assessing vagus nerve activity and reflexes. *Heart Fail. Rev.* **16**, 109–127 (2011). 20577901 10.1007/s10741-010-9174-6 PMC4322860

[16] Malik, M. Heart rate variability.: standards of measurement, physiological interpretation, and clinical use: task force of the European society of cardiology and the North American society for pacing and electrophysiology. *Ann. Noninv Electrocard*. **1**, 151–181 (1996).

[17] Shaffer, F. & Ginsberg, J. P. An overview of heart rate variability metrics and norms. *Front. Public. Health*. **5**, 258 (2017). 29034226 10.3389/fpubh.2017.00258 PMC5624990

[18] Gruber, J., Mennin, D. S., Fields, A., Purcell, A. & Murray, G. Heart rate variability as a potential indicator of positive Valence system disturbance: A proof of concept investigation. *Int. J. Psychophysiol.* **98**, 240–248 (2015). 26281850 10.1016/j.ijpsycho.2015.08.005

[19] Kogan, A., Gruber, J., Shallcross, A. J., Ford, B. Q. & Mauss, I. B. Too much of a good thing? Cardiac vagal tone’s nonlinear relationship with well-being. *Emotion* **13**, 599–604 (2013). 23731433 10.1037/a0032725

[20] Mccraty, R. & Shaffer, F. Heart rate variability: new perspectives on physiological mechanisms, assessment of Self-regulatory capacity, and health risk. *Glob Adv. Health Med.* **4**, 46–61 (2015). 25694852 10.7453/gahmj.2014.073 PMC4311559

[21] Thayer, J. F., Hansen, A. L., Saus-Rose, E. & Johnsen, B. H. Heart rate variability, prefrontal neural function, and cognitive performance: the neurovisceral integration perspective on Self-regulation, adaptation, and health. *ann. Behav. Med.* **37**, 141–153 (2009). 19424767 10.1007/s12160-009-9101-z

[22] Agren, T., Millroth, P., Andersson, P., Ridzén, M. & Björkstrand, J. Detailed analysis of skin conductance responses during a gambling task: decision, anticipation, and outcomes. *Psychophysiology* **56**, e13338 (2019). 30672602 10.1111/psyp.13338

[23] Mathar, D., Erfanian Abdoust, M., Marrenbach, T., Tuzsus, D. & Peters, J. The catecholamine precursor tyrosine reduces autonomic arousal and decreases decision thresholds in reinforcement learning and Temporal discounting. *PLoS Comput. Biol.* **18**, e1010785 (2022). 36548401 10.1371/journal.pcbi.1010785 PMC9822114

[24] Lempert, K. M., Johnson, E. & Phelps, E. A. Emotional arousal predicts intertemporal choice. *Emotion* **16**, 647–656 (2016). 26882337 10.1037/emo0000168 PMC4980249

[25] Ooteman, W., Koeter, M. W. J., Vserheul, R., Schippers, G. M. & Van Den Brink, W. Measuring craving: an attempt to connect subjective craving with cue reactivity. *Alcoholism Clin. Exp. Res.* **30**, 57–69 (2006). 10.1111/j.1530-0277.2006.00019.x 16433732

[26] Poláčková Šolcová, I. & Lačev, A. Differences in male and female subjective experience and physiological reactions to emotional stimuli. *Int. J. Psychophysiol.* **117**, 75–82 (2017). 28454989 10.1016/j.ijpsycho.2017.04.009

[27] Neale, C. et al. The impact of urban walking on Psychophysiological wellbeing. *Cities Health*. **6**, 1053–1066 (2022).

[28] Tost, H. et al. Neural correlates of individual differences in affective benefit of real-life urban green space exposure. *Nat. Neurosci.* **22**, 1389–1393 (2019). 31358990 10.1038/s41593-019-0451-y

[29] Van Den Berg, M. et al. Autonomic nervous system responses to viewing green and built settings: differentiating between sympathetic and parasympathetic activity. *IJERPH* **12**, 15860–15874 (2015). 26694426 10.3390/ijerph121215026 PMC4690962

[30] Wearne, T. A. et al. Anxiety sensitivity moderates the subjective experience but not the physiological response to psychosocial stress. *Int. J. Psychophysiol.* **141**, 76–83 (2019). 31054275 10.1016/j.ijpsycho.2019.04.012

[31] Ventura-Bort, C., Wendt, J. & Weymar, M. New insights on the correspondence between subjective affective experience and physiological responses from representational similarity analysis. *Psychophysiology* **59**, e14088 (2022). 35543530 10.1111/psyp.14088

[32] Berman, M. G., Jonides, J. & Kaplan, S. The cognitive benefits of interacting with nature. *Psychol. Sci.* **19**, 1207–1212 (2008). 19121124 10.1111/j.1467-9280.2008.02225.x

[33] Smith, T. W. et al. Matters of the variable heart: respiratory sinus arrhythmia response to marital interaction and associations with marital quality. *J. Personal. Soc. Psychol.* **100**, 103–119 (2011). 10.1037/a0021136 20954783

[34] Gevensleben, H. et al. Neurofeedback training in children with ADHD: 6-month follow-up of a randomised controlled trial. *Eur. Child. Adolesc. Psychiatry*. **19**, 715–724 (2010). 20499120 10.1007/s00787-010-0109-5 PMC3128749

[35] Kennedy, A. P. et al. Continuous in-the-field measurement of heart rate: correlates of drug use, craving, stress, and mood in polydrug users. *Drug Alcohol Depend.* **151**, 159–166 (2015). 25920802 10.1016/j.drugalcdep.2015.03.024 PMC4447529

[36] Bratman, G. N., Hamilton, J. P., Hahn, K. S., Daily, G. C. & Gross, J. J. Nature experience reduces rumination and subgenual prefrontal cortex activation. *Proc. Natl. Acad. Sci. USA*. **112**, 8567–8572 (2015). 26124129 10.1073/pnas.1510459112 PMC4507237

[37] Gidlow, C. J. et al. Where to put your best foot forward: Psycho-physiological responses to walking in natural and urban environments. *J. Environ. Psychol.* **45**, 22–29 (2016).

[38] Berman, M. G. et al. Interacting with nature improves cognition and affect for individuals with depression. *J. Affect. Disord.* **140**, 300–305 (2012). 22464936 10.1016/j.jad.2012.03.012 PMC3393816

[39] Kondo, M., Fluehr, J., McKeon, T. & Branas, C. Urban green space and its impact on human health. *IJERPH* **15**, 445 (2018). 29510520 10.3390/ijerph15030445 PMC5876990

[40] Roe, J. et al. The urban built environment, walking and mental health outcomes among older adults: A pilot study. *Front. Public. Health*. **8**, 575946 (2020). 33072714 10.3389/fpubh.2020.575946 PMC7538636

[41] Neale, C. et al. The impact of walking in different urban environments on brain activity in older people. *Cities Health*. **4**, 94–106 (2020).

[42] Hu, X. et al. From lab to life: evaluating the reliability and validity of Psychophysiological data from wearable devices in laboratory and ambulatory settings. *Behav. Res.*10.3758/s13428-024-02387-3 (2024). 10.3758/s13428-024-02387-3 PMC12403079 38528248

[43] Guijt, A. M., Sluiter, J. K. & Frings-Dresen, M. H. W. Test-Retest reliability of heart rate variability and respiration rate at rest and during light physical activity in normal subjects. *Arch. Med. Res.* **38**, 113–120 (2007). 17174734 10.1016/j.arcmed.2006.07.009

[44] Mathar, D., Wiebe, A., Tuzsus, D., Knauth, K. & Peters, J. Erotic cue exposure increases physiological arousal, biases choices toward immediate rewards, and attenuates model-based reinforcement learning. *Psychophysiology* **60**, e14381 (2023). 37435973 10.1111/psyp.14381

[45] Burma, J. S. et al. The validity and reliability of ultra-short-term heart rate variability parameters and the influence of physiological covariates. *J. Appl. Physiol.* **130**, 1848–1867 (2021). 33856258 10.1152/japplphysiol.00955.2020

[46] Mostajabi, J. & Johnson, S. L. Electrodermal lability and overt aggression: testing the trait-like nature of skin conductance responses. *Pers. Indiv. Differ.* **218**, 112475 (2024).

[47] Giuliani, N. P., Brown, C. J. & Wu, Y-H. Comparisons of the sensitivity and reliability of multiple measures of listening effort. *Ear Hear.* **42**, 465–474 (2021). 32925306 10.1097/AUD.0000000000000950 PMC9135174

[48] Borrego, A., Latorre, J., Alcaniz, M. & Llorens, R. Reliability of the Empatica E4 wristband to measure electrodermal activity to emotional stimuli. In: 2019 International Conference on Virtual Rehabilitation (ICVR). IEEE, Tel Aviv, Israel, pp 1–2 (2019).

[49] Carrier, B., Barrios, B., Jolley, B. D. & Navalta, J. W. Validity and reliability of physiological data in applied settings measured by wearable technology: A rapid. *Syst. Rev. Technol.* **8**, 70 (2020).

[50] Pratiwi, P. I., Xiang, Q. & Furuya, K. Physiological and psychological effects of viewing urban parks in different seasons in adults. *IJERPH* **16**, 4279 (2019). 31689960 10.3390/ijerph16214279 PMC6862170

[51] Xiang, Q., Yuan, Z. & Mao, Y. Psychological and physiological effects in Satoyama activities of older adult volunteers in the urban green space in spring and autumn. *J. Psychiatry Psychiatric Disorders*. **07**, 60–79 (2023).

[52] Montes, J., Young, J. C., Tandy, R. & Navalta, J. W. *Reliability and Validation of the Hexoskin Wearable* (Bio-Collection Device During Walking Conditions, 2018). 10.70252/YPHF4748 PMC6179424 30338022

[53] Reichert, M. et al. Ambulatory assessment for precision psychiatry: foundations, current developments and future avenues. *Exp. Neurol.* **345**, 113807 (2021). 34228998 10.1016/j.expneurol.2021.113807

[54] Riesel, A., Endrass, T. & Weinberg, A. Biomarkers of mental disorders: Psychophysiological measures as indicators of mechanisms, risk, and outcome prediction. *Int. J. Psychophysiol.* **168**, 21–26 (2021). 34364039 10.1016/j.ijpsycho.2021.06.014

[55] Dudarev, V., Barral, O., Zhang, C., Davis, G. & Enns, J. T. On the reliability of wearable technology: A tutorial on measuring heart rate and heart rate variability in the wild. *Sensors* **23**, 5863 (2023). 37447713 10.3390/s23135863 PMC10346338

[56] Tavakol, M. & Dennick, R. Making sense of cronbach’s alpha. *Int. J. Med. Educ.* **2**, 53–55 (2011). 28029643 10.5116/ijme.4dfb.8dfd PMC4205511

[57] Babbie, E. R. The practice of social research, Fourteenth edition. Cengage Learning, Boston, MA (2016).

[58] Clifton, J. D. W. Managing validity versus reliability trade-offs in scale-building decisions. *Psychol. Methods*. **25**, 259–270 (2020). 31414848 10.1037/met0000236

[59] McKinnon, J. Reliability and validity in field research: some strategies and tactics. *Acc. Auditing Account. J.* **1**, 34–54 (1988).

[60] Mestdagh, M. & Dejonckheere, E. Ambulatory assessment in psychopathology research: current achievements and future ambitions. *Curr. Opin. Psychol.* **41**, 1–8 (2021). 33550191 10.1016/j.copsyc.2021.01.004

[61] Berthoud, H-R. Multiple neural systems controlling food intake and body weight. *Neurosci. Biobehavioral Reviews*. **26**, 393–428 (2002). 10.1016/s0149-7634(02)00014-3 12204189

[62] Schwerdtfeger, A. R. & Rominger, C. Acute fasting modulates autonomic nervous system function and ambulatory cardiac interoception. *Biol. Psychol.* **186**, 108760 (2024). 38331345 10.1016/j.biopsycho.2024.108760

[63] Herbert, B. M. et al. Effects of short-term food deprivation on interoceptive awareness, feelings and autonomic cardiac activity. *Biol. Psychol.* **89**, 71–79 (2012). 21958594 10.1016/j.biopsycho.2011.09.004

[64] Flasbeck, V., Bamberg, C. & Brüne, M. Short-Term fasting and ingestion of caloric drinks affect Heartbeat-Evoked potentials and autonomic nervous system activity in males. *Front. Neurosci.* **15**, 622428 (2021). 34267619 10.3389/fnins.2021.622428 PMC8276132

[65] Gonzalez, J. E. & Cooke, W. H. Acute fasting reduces tolerance to progressive central hypovolemia in humans. *J. Appl. Physiol.* **136**, 362–371 (2024). 38126086 10.1152/japplphysiol.00622.2023 PMC11219002

[66] Faul, F., Erdfelder, E., Buchner, A. & Lang, A-G. Statistical power analyses using G* power 3.1: tests for correlation and regression analyses. *Behav. Res. Methods*. **41**, 1149–1160 (2009). 19897823 10.3758/BRM.41.4.1149

[67] Movisens, G. H. ECGMove 4 EdaMove 4. (2020).

[68] Posit Team. RStudio: Integrated Development Environment for R. Posit, PBC (2024). Available at:

[69] The MathWorks, Inc. MATLAB version 2019a. (2022).

[70] Dawson ME, Schell AM, Filion DL. The electrodermal system. In: Cacioppo JT, Tassinary LG, Berntson GG (eds.) *Handbook of Psychophysiology*, 3rd edn, 159–181. Cambridge University Press, Cambridge (2007).

[71] Hamilton P. Open source ECG analysis. *Comput. Cardiol*. 101–104 (2002).

[72] Clifford GD, McSharry PE, Tarassenko L. Characterizing artefact in the normal human 24-hour RR time series to aid identification and artificial replication of circadian variations in human beat to beat heart rate using a simple threshold. In: *Comp. Cardiol*. 129–132. 10.1109/CIC.2002.1166724 (2002).

[73] Wehler, D. et al. Reliability of heart-rate-variability features derived from ultra-short ECG recordings and their validity in the assessment of cardiac autonomic neuropathy. *Biomed. Signal Process. Control*. **68**, 102651 (2021).

[74] Wilhelm, F. H. & Grossman, P. Emotions beyond the laboratory: theoretical fundaments, study design, and analytic strategies for advanced ambulatory assessment. *Biol. Psychol.* **84**, 552–569 (2010). 20132861 10.1016/j.biopsycho.2010.01.017

[75] Lever, J., Krzywinski, M. & Altman, N. Principal component analysis. *Nat. Methods*. **14**, 641–642 (2017).

[76] Wright, D. B., London, K. & Field, A. P. Using bootstrap Estimation and the Plug-in principle for clinical psychology data. *J. Experimental Psychopathol.* **2**, 252–270 (2011).

[77] Parsons, S., Kruijt, A-W. & Fox, E. Psychological science needs a standard practice of reporting the reliability of Cognitive-Behavioral measurements. *Adv. Methods Practices Psychol. Sci.* **2**, 378–395 (2019).

[78] Koo, T. K. & Li, M. Y. A guideline of selecting and reporting intraclass correlation coefficients for reliability research. *J. Chiropr. Med.* **15**, 155–163 (2016). 27330520 10.1016/j.jcm.2016.02.012 PMC4913118

[79] Kuckartz, U., Rädiker, S., Ebert, T. & Schehl, J. Statistik: Eine verständliche Einführung. (2013). 10.1007/978-3-531-19890-3

[80] Rammstedt, B. Zur Bestimmung der Güte von Multi-Item-Skalen: Eine Einführung. (2004).

[81] Nunnally, J. C. & Bernstein, I. H. *Psychometric Theory* 3rd edn (McGraw-Hill, 1994).

[82] Weise, G. *Psychologische Leistungstests* (Hogrefe, 1975).

[83] Rodebaugh, T. L. et al. Unreliability as a threat to Understanding psychopathology: the cautionary Tale of attentional bias. *J. Abnorm. Psychol.* **125**, 840–851 (2016). 27322741 10.1037/abn0000184 PMC4980228

[84] Kline, P. The handbook of psychological testing, 2nd ed. vii, 744 (1993).

[85] OpenStreetMap contributors OpenStreetMap.

[86] Cheng, J., Karambelkar, B. & Xie, Y. leaflet: Create Interactive Web Maps with the JavaScript Leaflet Library. (2023).

[87] Agafonkin, V. Leaflet: An open-source JavaScript library for mobile-friendly interactive maps. (2011).

[88] Ainsworth, B. E. et al. 2011 compendium of physical activities: a second update of codes and MET values. *Med. Sci. Sports Exerc.* **43**, 1575–1581 (2011). 21681120 10.1249/MSS.0b013e31821ece12

[89] Riebe, D., Ehrman, J. K., Liguori, G. & Magal, M. *ACSM’s Guidelines for Exercise Testing and Prescription* (American College of Sports Medicine, 2018).

[90] Kazmi, S. Z. H. et al. Inverse correlation between heart rate variability and heart rate demonstrated by linear and nonlinear analysis. *PLoS ONE*. **11**, e0157557 (2016). 27336907 10.1371/journal.pone.0157557 PMC4919077

[91] Geršak, G. Electrodermal activity - a beginner’s guide. *Electrotechnical Rev.* **87**, 175–182 (2020).

[92] Jolliffe, I. T. *Principal Component Analysis for Special Types of Data* (Springer, 2002).

[93] Strang, G. Linear algebra and its applications. (2000).

[94] Siegel, S., Castellan, N. J. & Castellan, N. J. *Nonparametric Statistics for the Behavioral Sciences* 2. edn (MacGraw-Hill, 1988).

[95] Johnson, K. T. & Picard, R. W. Advancing neuroscience through wearable devices. *Neuron* **108**, 8–12 (2020). 33058768 10.1016/j.neuron.2020.09.030

[96] Houtveen, J. H. & de Geus, E. J. C. Noninvasive Psychophysiological ambulatory recordings: study design and data analysis strategies. *Eur. Psychol.* **14**, 132–141 (2009).

[97] Sterling P, Eyer J. Allostasis: a new paradigm to explain arousal pathology. In: Fisher S, Reason J (eds.) *Handbook of Life Stress, Cognition and Health*, 629–649. John Wiley & Sons, New York (1988).

[98] Babisch, W. Guest editorial: noise and health. *Environ. Health Perspect.*10.1289/ehp.113-a14 (2005). 15631951 10.1289/ehp.113-a14 PMC1253720

[99] Shiffrin, R. M. & Schneider, W. Controlled and Automatic Human Information Processing: II. Perceptual Learning, Automatic Attending, and a General Theory.

[100] Ingjaldsson, J. T., Laberg, J. C. & Thayer, J. F. Reduced heart rate variability in chronic alcohol abuse: relationship with negative mood, chronic thought suppression, and compulsive drinking. *Biol. Psychiatry*. **54**, 1427–1436 (2003). 14675808 10.1016/s0006-3223(02)01926-1

